# Tris[4,4,4-trifluoro-1-(2-thien­yl)butane-1,3-dionato]aluminium(III)–tris­[4,4,4-trifluoro-1-(2-thien­yl)butane-1,3-dion­ato]iron(III) (3/1)

**DOI:** 10.1107/S160053680902858X

**Published:** 2009-07-25

**Authors:** Madeleine Schultz, Elnor C. Roa

**Affiliations:** a School of Physical and Chemical Sciences, Queensland University of Technology, Brisbane, Queensland 4001, Australia; b Chemistry Department, College of Sciences and Mathematics, Mindanao State University - Iligan Institute of Technology, Iligan City, Philippines

## Abstract

In the title compound, [Al(C_8_H_4_F_3_O_2_S)_3_]_3_[Fe(C_8_H_4_F_3_O_2_S)_3_], the metal centre is statistically occupied by Al^III^ and Fe^III^ cations in a 3:1 ratio. The metal centre is within an octa­hedral O_6_ donor set defined by three chelating substituted acetoacetonate anions. The ligands are arranged around the periphery of the mol­ecule with a *mer* geometry of the S atoms.

## Related literature

The analogous structures of the octa­hedral Fe, In (Soling, 1976 ▶) and Ru (Aynetchi *et al.*, 1986 ▶) complexes of this ligand have been reported. For extraction with supercritical carbon dioxide using this ligand, see: Wai (1995 ▶); Wai *et al.* (1996 ▶). For related Al acetyl­acetonato structures, see: Bott *et al.* (2001 ▶); Dharmaprakash *et al.* (2006 ▶).
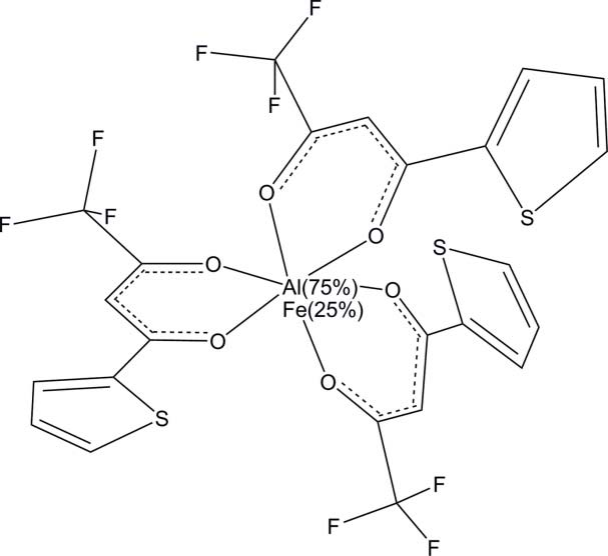


## Experimental

### 

#### Crystal data

[Al(C_8_H_4_F_3_O_2_S)_3_]_3_[Fe(C_8_H_4_F_3_O_2_S)_3_]
*M* *_r_* = 2790.97
Monoclinic, 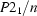

*a* = 13.743 (3) Å
*b* = 14.278 (2) Å
*c* = 14.136 (3) Å
β = 94.013 (18)°
*V* = 2767.1 (9) Å^3^
*Z* = 1
Mo *K*α radiation
μ = 0.52 mm^−1^
*T* = 173 K
0.14 × 0.10 × 0.10 mm

#### Data collection

Oxford Diffraction Xcalibur diffractometer with a Sapphire3 detector
Absorption correction: none
11706 measured reflections
6021 independent reflections
2687 reflections with *I* > 2σ(*I*)
*R*_int_ = 0.052

#### Refinement

*R*[*F* ^2^ > 2σ(*F* ^2^)] = 0.071
*wR*(*F* ^2^) = 0.185
*S* = 0.91
6021 reflections
388 parameters
H-atom parameters constrained
Δρ_max_ = 0.72 e Å^−3^
Δρ_min_ = −0.44 e Å^−3^

### 

Data collection: *CrysAlis Pro* (Oxford Diffraction, 2009 ▶); cell refinement: *CrysAlis Pro*; data reduction: *CrysAlis Pro*; program(s) used to solve structure: *SIR92* (Altomare *et al.*, 1994 ▶); program(s) used to refine structure: *SHELXL97* (Sheldrick, 2008 ▶); molecular graphics: *ORTEP-3* (Farrugia, 1997 ▶) and *WinGX32* (Farrugia, 1999 ▶); software used to prepare material for publication: *SHELXL97*.

## Supplementary Material

Crystal structure: contains datablocks I, global. DOI: 10.1107/S160053680902858X/tk2491sup1.cif

Structure factors: contains datablocks I. DOI: 10.1107/S160053680902858X/tk2491Isup2.hkl

Additional supplementary materials:  crystallographic information; 3D view; checkCIF report

## Figures and Tables

**Fig. 1.**
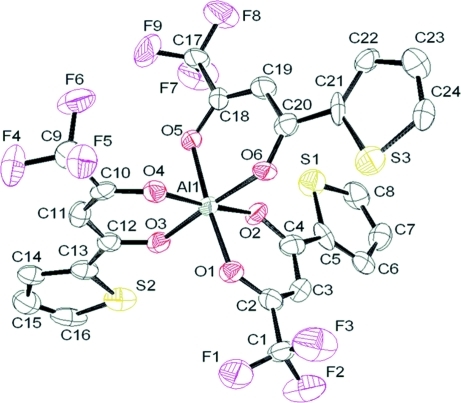
A view of the complex (I) showing the atom labelling scheme. Hydrogen atoms have been omitted for clarity and displacement ellipsoids are drawn at the 50% probability level. The Al1 site is 25% occupied by Fe.

## supplementary crystallographic information

### Comment

The title compound (I) was formed during the extraction of a standard reference material using supercritical carbon dioxide (SFE) in order to determine the recovery of some heavy metals by ICP-MS. SFE using fluorinated ligands such as 1-(2-thienyl)-4,4,4-trifluoro-1,3-butanedione has been shown to be very effective in extracting metals from a variety of matrices (Wai, 1995; Wai *et al.*, 1996).

The structure of (I), Fig. 1, is similar to those of the Ru (Aynetchi *et al.*, 1986), In and Fe (Soling, 1976) complexes of the same ligand. As observed in those structures, the M—O distances adjacent to the trifluoromethyl group are very slightly shorter than those  adjacent to the thienyl group. A more important bond distance variation is found in the backbone of the butanedionato ligand, in that the two C—C bond distances in the chelate ring are significantly different from each other. The distance from the central C atom of the chelate ring to the C atom adjacent to the trifluoromethyl group (C2—C3; C10—C11; C18—C19) is significantly shorter than that to the C atom adjacent to the thienyl group (C3—C4; C11—C12; C19—C20). In the present structure this difference is around 0.06 Å, which is similar to that observed in the three reported structures involving octahedral complexes of this ligand.

A large number of structures of Al(acac)_3_ have been reported with the unsubstituted acetylacetonato ligand (Bott *et al.*, 2001), and the related complex *tris*(3-t-butoxybutandionato)aluminium(III), bearing a heterobidentate acetylacetonato ligand has also been reported (Dharmaprakash *et al.*, 2006). In that structure, a *fac* arrangement of the ligands was observed, unlike in the present structure.

### Experimental

A sample of a standard reference material NCSDC 73372, Lake Sediment, was extracted using supercritical CO_2_ modified with 5% methanol in the presence of the protonated ligand 1-(2-thienyl)-4,4,4-trifluoro- 1,3-butanedione (Wai, 1995; Wai *et al.*, 1996). The extract was collected in chloroform and the deep-red crystals formed upon evaporation of the solvent. The identity of the metal was determined after the crystallographic data had been collected by subjecting the same crystal to Scanning Electron Microscopy coupled with Energy Dispersive X-ray spectroscopy (SEM-EDX). The spectra obtained at two different locations on the crystal showed the only metals present in detectable amounts to be Al and Fe. The ratio of these was found to be 3:1 by integrating the K peaks after background subtraction.

### Refinement

All H atoms were positioned geometrically and allowed to ride on their parent atoms with C—H distances of 0.95 Å, and with *U*_iso_(H) = 1.2 *U*_eq_(C).

There is gross thermal motion in one trifluoromethyl group of one ligand, affecting the thermal parameters of F7, F8 and F9, but this does not adversely impact the quality of the core of the structure. It is interesting to examine the residual electron density in detail; the thienyl groups appear to have very low occupancy in which the sulfur atom lies on the opposite side of the planar thienyl ring. This leads to some anomalies in the thermal parameters in those rings. However, all bond distances and angles fall within normal ranges.

### Crystal data

**Table:**

[Al(C_8_H_4_F_3_O_2_S)_3_]_3_[Fe(C_8_H_4_F_3_O_2_S)_3_]	*F*(000) = 1397
*M**_r_* = 2790.97	*D*_x_ = 1.675 Mg m^−^^3^
Monoclinic, *P*2_1_/*n*	Mo *K*α radiation, λ = 0.71073 Å
Hall symbol: -P 2yn	Cell parameters from 1790 reflections
*a* = 13.743 (3) Å	θ = 2.9–28.0°
*b* = 14.278 (2) Å	µ = 0.52 mm^−^^1^
*c* = 14.136 (3) Å	*T* = 173 K
β = 94.013 (18)°	Block, red
*V* = 2767.1 (9) Å^3^	0.14 × 0.10 × 0.10 mm
*Z* = 1	

### Data collection

**Table:**

Oxford Diffraction Xcalibur diffractometer with a Sapphire3 (Gemini Ultra Mo) detector	2687 reflections with *I* > 2σ(*I*)
Radiation source: fine-focus sealed tube	*R*_int_ = 0.052
graphite	θ_max_ = 28.0°, θ_min_ = 2.9°
ω scans	*h* = −18→14
11706 measured reflections	*k* = −16→18
6021 independent reflections	*l* = −13→18

### Refinement

**Table:**

Refinement on *F*^2^	Primary atom site location: structure-invariant direct methods
Least-squares matrix: full	Secondary atom site location: difference Fourier map
*R*[*F*^2^ > 2σ(*F*^2^)] = 0.071	Hydrogen site location: inferred from neighbouring sites
*wR*(*F*^2^) = 0.185	H-atom parameters constrained
*S* = 0.91	*w* = 1/[σ^2^(*F*_o_^2^) + (0.0829*P*)^2^] where *P* = (*F*_o_^2^ + 2*F*_c_^2^)/3
6021 reflections	(Δ/σ)_max_ < 0.001
388 parameters	Δρ_max_ = 0.72 e Å^−^^3^
0 restraints	Δρ_min_ = −0.44 e Å^−^^3^

### Special details

**Table:**

Geometry. All e.s.d.'s (except the e.s.d. in the dihedral angle between two l.s. planes) are estimated using the full covariance matrix. The cell e.s.d.'s are taken into account individually in the estimation of e.s.d.'s in distances, angles and torsion angles; correlations between e.s.d.'s in cell parameters are only used when they are defined by crystal symmetry. An approximate (isotropic) treatment of cell e.s.d.'s is used for estimating e.s.d.'s involving l.s. planes.
Refinement. Refinement of *F*^2^ against ALL reflections. The weighted *R*-factor *wR* and goodness of fit *S* are based on *F*^2^, conventional *R*-factors *R* are based on *F*, with *F* set to zero for negative *F*^2^. The threshold expression of *F*^2^ > σ(*F*^2^) is used only for calculating *R*-factors(gt) *etc*. and is not relevant to the choice of reflections for refinement. *R*-factors based on *F*^2^ are statistically about twice as large as those based on *F*, and *R*- factors based on ALL data will be even larger.

### Fractional atomic coordinates and isotropic or equivalent isotropic displacement parameters (Å^2^)

**Table:**

	*x*	*y*	*z*	*U*_iso_*/*U*_eq_	Occ. (<1)
Al1	0.69004 (9)	0.19827 (9)	0.06179 (8)	0.0295 (3)	0.75
Fe1	0.69004 (9)	0.19827 (9)	0.06179 (8)	0.0295 (3)	0.25
S3	0.44342 (12)	0.41369 (12)	0.13998 (12)	0.0543 (5)	
S2	0.89747 (12)	−0.06384 (12)	0.11705 (12)	0.0585 (5)	
S1	0.85479 (12)	0.30057 (13)	0.34316 (12)	0.0563 (5)	
F5	0.5874 (2)	0.1933 (2)	−0.2505 (2)	0.0519 (9)	
O3	0.7705 (2)	0.0890 (2)	0.0564 (2)	0.0360 (9)	
F4	0.7125 (3)	0.1207 (3)	−0.2951 (2)	0.0676 (11)	
O5	0.7945 (2)	0.2798 (2)	0.0298 (2)	0.0335 (9)	
O2	0.7411 (2)	0.2155 (3)	0.1911 (2)	0.0367 (9)	
F8	0.8943 (3)	0.5020 (3)	0.0181 (3)	0.0840 (14)	
O1	0.5872 (2)	0.1250 (2)	0.1022 (2)	0.0344 (9)	
O6	0.6072 (2)	0.3059 (2)	0.0761 (2)	0.0336 (9)	
F2	0.4760 (3)	0.0067 (3)	0.2755 (3)	0.0831 (13)	
F3	0.3977 (3)	0.1018 (3)	0.1898 (3)	0.0889 (14)	
F1	0.4669 (3)	−0.0117 (3)	0.1286 (3)	0.0901 (15)	
F7	0.9645 (3)	0.3814 (3)	0.0733 (3)	0.0868 (14)	
F6	0.7253 (3)	0.2621 (3)	−0.2414 (2)	0.0709 (12)	
O4	0.6526 (2)	0.1881 (2)	−0.0684 (2)	0.0334 (9)	
C12	0.8008 (4)	0.0479 (4)	−0.0163 (4)	0.0337 (13)	
F9	0.9159 (3)	0.3810 (3)	−0.0685 (3)	0.0972 (16)	
C14	0.9116 (4)	−0.0882 (4)	−0.0661 (4)	0.0377 (14)	
H14	0.9026	−0.0827	−0.1331	0.045*	
C18	0.7963 (4)	0.3683 (4)	0.0399 (4)	0.0372 (14)	
C10	0.7039 (4)	0.1449 (4)	−0.1282 (3)	0.0312 (13)	
C2	0.5671 (4)	0.1104 (4)	0.1884 (4)	0.0414 (15)	
C9	0.6824 (4)	0.1807 (4)	−0.2294 (4)	0.0445 (15)	
C22	0.5581 (4)	0.5545 (4)	0.1173 (4)	0.0389 (14)	
H22	0.6125	0.5926	0.1053	0.047*	
C3	0.6194 (4)	0.1384 (4)	0.2702 (4)	0.0409 (14)	
H3	0.5975	0.1198	0.3297	0.049*	
C21	0.5569 (4)	0.4602 (4)	0.1117 (3)	0.0396 (15)	
C6	0.7347 (4)	0.2167 (4)	0.4512 (4)	0.0355 (13)	
H6	0.6831	0.1803	0.4733	0.043*	
C13	0.8660 (4)	−0.0308 (4)	0.0018 (4)	0.0416 (14)	
C1	0.4768 (4)	0.0521 (4)	0.1955 (4)	0.0428 (15)	
C24	0.4008 (4)	0.5220 (4)	0.1584 (4)	0.0459 (16)	
H24	0.3371	0.5348	0.1773	0.055*	
C4	0.7053 (4)	0.1944 (4)	0.2686 (4)	0.0395 (14)	
C7	0.8050 (5)	0.2679 (5)	0.5097 (4)	0.0498 (16)	
H7	0.8054	0.2694	0.5769	0.060*	
C11	0.7736 (4)	0.0785 (4)	−0.1096 (4)	0.0362 (13)	
H11	0.8050	0.0518	−0.1610	0.043*	
C17	0.8902 (4)	0.4101 (4)	0.0143 (5)	0.0466 (16)	
C19	0.7239 (4)	0.4262 (4)	0.0665 (4)	0.0447 (15)	
H19	0.7371	0.4912	0.0738	0.054*	
C8	0.8712 (5)	0.3141 (4)	0.4601 (4)	0.0563 (18)	
H8	0.9224	0.3509	0.4896	0.068*	
C23	0.4676 (5)	0.5887 (5)	0.1434 (4)	0.0568 (18)	
H23	0.4546	0.6536	0.1498	0.068*	
C20	0.6284 (4)	0.3921 (4)	0.0839 (3)	0.0366 (14)	
C5	0.7541 (4)	0.2285 (4)	0.3563 (4)	0.0356 (14)	
C15	0.9723 (5)	−0.1543 (5)	−0.0158 (5)	0.0576 (19)	
H15	1.0115	−0.1979	−0.0468	0.069*	
C16	0.9703 (4)	−0.1506 (4)	0.0786 (6)	0.061 (2)	
H16	1.0061	−0.1924	0.1203	0.073*	

### Atomic displacement parameters (Å^2^)

**Table:**

	*U*^11^	*U*^22^	*U*^33^	*U*^12^	*U*^13^	*U*^23^
Al1	0.0308 (7)	0.0283 (7)	0.0301 (7)	−0.0074 (6)	0.0079 (5)	−0.0021 (6)
Fe1	0.0308 (7)	0.0283 (7)	0.0301 (7)	−0.0074 (6)	0.0079 (5)	−0.0021 (6)
S3	0.0459 (10)	0.0551 (11)	0.0631 (11)	−0.0076 (8)	0.0130 (8)	−0.0013 (9)
S2	0.0528 (11)	0.0485 (11)	0.0735 (12)	−0.0058 (9)	0.0003 (8)	0.0154 (9)
S1	0.0486 (10)	0.0621 (12)	0.0582 (11)	−0.0068 (9)	0.0037 (8)	−0.0030 (9)
F5	0.047 (2)	0.062 (2)	0.045 (2)	0.0062 (19)	−0.0039 (15)	−0.0006 (17)
O3	0.040 (2)	0.034 (2)	0.034 (2)	−0.0012 (18)	0.0059 (17)	0.0066 (18)
F4	0.071 (3)	0.094 (3)	0.039 (2)	0.028 (2)	0.0096 (18)	−0.008 (2)
O5	0.031 (2)	0.032 (2)	0.039 (2)	−0.0064 (17)	0.0133 (16)	−0.0031 (18)
O2	0.034 (2)	0.046 (3)	0.031 (2)	−0.0091 (18)	0.0102 (16)	0.0005 (18)
F8	0.052 (3)	0.046 (3)	0.160 (4)	−0.016 (2)	0.050 (3)	−0.004 (2)
O1	0.034 (2)	0.037 (2)	0.032 (2)	−0.0125 (18)	0.0002 (16)	−0.0015 (18)
O6	0.038 (2)	0.026 (2)	0.037 (2)	−0.0130 (18)	0.0047 (16)	−0.0020 (17)
F2	0.088 (3)	0.084 (3)	0.076 (3)	−0.035 (3)	−0.002 (2)	0.025 (2)
F3	0.035 (2)	0.074 (3)	0.158 (4)	0.001 (2)	0.007 (2)	0.018 (3)
F1	0.096 (3)	0.083 (3)	0.095 (3)	−0.062 (3)	0.033 (2)	−0.039 (3)
F7	0.042 (2)	0.084 (3)	0.133 (4)	−0.020 (2)	−0.005 (2)	0.006 (3)
F6	0.085 (3)	0.071 (3)	0.057 (2)	−0.036 (2)	0.005 (2)	0.018 (2)
O4	0.031 (2)	0.031 (2)	0.039 (2)	−0.0044 (17)	0.0050 (16)	−0.0017 (18)
C12	0.026 (3)	0.030 (3)	0.046 (4)	−0.009 (2)	0.006 (2)	0.000 (3)
F9	0.106 (3)	0.096 (3)	0.099 (3)	−0.051 (3)	0.078 (3)	−0.027 (3)
C14	0.022 (3)	0.032 (3)	0.060 (4)	−0.003 (3)	0.004 (3)	0.004 (3)
C18	0.043 (3)	0.035 (4)	0.036 (3)	−0.005 (3)	0.013 (3)	−0.006 (3)
C10	0.026 (3)	0.035 (3)	0.032 (3)	−0.018 (3)	−0.001 (2)	0.004 (3)
C2	0.042 (4)	0.032 (3)	0.051 (4)	−0.009 (3)	0.009 (3)	−0.008 (3)
C9	0.038 (4)	0.052 (4)	0.044 (4)	−0.003 (3)	0.002 (3)	−0.001 (3)
C22	0.033 (3)	0.046 (4)	0.040 (3)	−0.013 (3)	0.014 (3)	−0.001 (3)
C3	0.043 (4)	0.043 (4)	0.038 (3)	−0.002 (3)	0.015 (3)	0.000 (3)
C21	0.046 (4)	0.053 (4)	0.020 (3)	0.025 (3)	0.004 (2)	−0.012 (3)
C6	0.025 (3)	0.028 (3)	0.053 (4)	0.010 (2)	−0.003 (3)	0.000 (3)
C13	0.032 (3)	0.036 (3)	0.057 (4)	−0.013 (3)	0.002 (3)	0.005 (3)
C1	0.044 (4)	0.041 (4)	0.045 (4)	−0.016 (3)	0.010 (3)	0.005 (3)
C24	0.041 (4)	0.052 (4)	0.046 (4)	0.008 (3)	0.017 (3)	−0.006 (3)
C4	0.034 (3)	0.033 (3)	0.051 (4)	0.005 (3)	0.002 (3)	0.011 (3)
C7	0.052 (4)	0.062 (4)	0.035 (3)	0.010 (3)	0.004 (3)	0.005 (3)
C11	0.029 (3)	0.047 (4)	0.032 (3)	−0.003 (3)	0.003 (2)	−0.005 (3)
C17	0.038 (4)	0.032 (4)	0.073 (5)	−0.019 (3)	0.021 (3)	−0.007 (3)
C19	0.039 (3)	0.035 (3)	0.062 (4)	−0.014 (3)	0.015 (3)	−0.005 (3)
C8	0.047 (4)	0.054 (4)	0.067 (4)	−0.012 (3)	−0.006 (3)	−0.022 (4)
C23	0.070 (5)	0.043 (4)	0.058 (4)	−0.001 (4)	0.005 (4)	−0.013 (3)
C20	0.039 (3)	0.043 (4)	0.027 (3)	−0.009 (3)	0.001 (2)	0.000 (3)
C5	0.031 (3)	0.031 (3)	0.042 (3)	0.017 (3)	−0.019 (2)	−0.014 (3)
C15	0.048 (4)	0.043 (4)	0.084 (5)	−0.003 (3)	0.020 (4)	−0.005 (4)
C16	0.040 (4)	0.031 (4)	0.111 (6)	−0.001 (3)	−0.001 (4)	0.025 (4)

### Geometric parameters (Å, °)

**Table:**

Al1—O1	1.879 (3)	C14—C13	1.438 (7)
Al1—O4	1.882 (4)	C14—H14	0.9500
Al1—O3	1.918 (4)	C18—C19	1.368 (7)
Al1—O5	1.927 (3)	C18—C17	1.489 (7)
Al1—O2	1.928 (4)	C10—C11	1.359 (7)
Al1—O6	1.932 (4)	C10—C9	1.528 (7)
S3—C24	1.680 (6)	C2—C3	1.378 (7)
S3—C21	1.767 (6)	C2—C1	1.503 (7)
S2—C16	1.706 (7)	C22—C21	1.348 (7)
S2—C13	1.722 (6)	C22—C23	1.408 (8)
S1—C8	1.664 (6)	C22—H22	0.9500
S1—C5	1.744 (6)	C3—C4	1.428 (7)
F5—C9	1.331 (6)	C3—H3	0.9500
O3—C12	1.277 (6)	C21—C20	1.456 (7)
F4—C9	1.349 (6)	C6—C5	1.396 (7)
O5—C18	1.271 (6)	C6—C7	1.427 (8)
O2—C4	1.267 (6)	C6—H6	0.9500
F8—C17	1.314 (6)	C24—C23	1.351 (8)
O1—C2	1.284 (6)	C24—H24	0.9500
O6—C20	1.267 (6)	C4—C5	1.452 (7)
F2—C1	1.305 (6)	C7—C8	1.359 (8)
F3—C1	1.296 (7)	C7—H7	0.9500
F1—C1	1.313 (6)	C11—H11	0.9500
F7—C17	1.337 (7)	C19—C20	1.437 (7)
F6—C9	1.319 (6)	C19—H19	0.9500
O4—C10	1.295 (6)	C8—H8	0.9500
C12—C11	1.415 (7)	C23—H23	0.9500
C12—C13	1.449 (8)	C15—C16	1.338 (8)
F9—C17	1.314 (6)	C15—H15	0.9500
C14—C15	1.418 (8)	C16—H16	0.9500
			
O1—Al1—O4	95.39 (15)	C20—C21—S3	115.6 (5)
O1—Al1—O3	90.41 (15)	C5—C6—C7	109.1 (5)
O4—Al1—O3	90.98 (15)	C5—C6—H6	125.4
O1—Al1—O5	175.17 (16)	C7—C6—H6	125.4
O4—Al1—O5	88.53 (15)	C14—C13—C12	128.1 (5)
O3—Al1—O5	92.33 (15)	C14—C13—S2	112.5 (4)
O1—Al1—O2	90.83 (14)	C12—C13—S2	119.4 (4)
O4—Al1—O2	173.74 (15)	F3—C1—F2	105.5 (5)
O3—Al1—O2	88.24 (15)	F3—C1—F1	106.9 (5)
O5—Al1—O2	85.29 (15)	F2—C1—F1	105.8 (5)
O1—Al1—O6	87.13 (15)	F3—C1—C2	112.7 (5)
O4—Al1—O6	92.32 (15)	F2—C1—C2	113.0 (5)
O3—Al1—O6	176.06 (14)	F1—C1—C2	112.3 (5)
O5—Al1—O6	89.90 (15)	C23—C24—S3	112.1 (4)
O2—Al1—O6	88.71 (15)	C23—C24—H24	123.9
C24—S3—C21	90.7 (3)	S3—C24—H24	123.9
C16—S2—C13	90.7 (3)	O2—C4—C3	121.2 (5)
C8—S1—C5	90.9 (3)	O2—C4—C5	118.3 (5)
C12—O3—Al1	128.8 (3)	C3—C4—C5	120.5 (5)
C18—O5—Al1	125.6 (3)	C8—C7—C6	113.6 (5)
C4—O2—Al1	130.7 (4)	C8—C7—H7	123.2
C2—O1—Al1	126.5 (3)	C6—C7—H7	123.2
C20—O6—Al1	130.4 (3)	C10—C11—C12	122.2 (5)
C10—O4—Al1	123.4 (3)	C10—C11—H11	118.9
O3—C12—C11	121.9 (5)	C12—C11—H11	118.9
O3—C12—C13	116.5 (5)	F9—C17—F8	109.9 (5)
C11—C12—C13	121.6 (5)	F9—C17—F7	102.6 (5)
C15—C14—C13	108.3 (5)	F8—C17—F7	104.6 (5)
C15—C14—H14	125.9	F9—C17—C18	112.4 (5)
C13—C14—H14	125.9	F8—C17—C18	115.2 (5)
O5—C18—C19	128.6 (5)	F7—C17—C18	111.2 (5)
O5—C18—C17	112.5 (5)	C18—C19—C20	122.2 (5)
C19—C18—C17	118.9 (5)	C18—C19—H19	118.9
O4—C10—C11	127.9 (5)	C20—C19—H19	118.9
O4—C10—C9	111.9 (5)	C7—C8—S1	114.0 (5)
C11—C10—C9	120.1 (5)	C7—C8—H8	123.0
O1—C2—C3	128.0 (5)	S1—C8—H8	123.0
O1—C2—C1	112.8 (5)	C24—C23—C22	114.8 (6)
C3—C2—C1	119.2 (5)	C24—C23—H23	122.6
F6—C9—F5	106.9 (5)	C22—C23—H23	122.6
F6—C9—F4	108.1 (5)	O6—C20—C19	121.4 (5)
F5—C9—F4	105.9 (5)	O6—C20—C21	121.2 (5)
F6—C9—C10	110.8 (5)	C19—C20—C21	117.4 (5)
F5—C9—C10	112.2 (4)	C6—C5—C4	132.3 (5)
F4—C9—C10	112.5 (5)	C6—C5—S1	112.3 (4)
C21—C22—C23	110.8 (5)	C4—C5—S1	115.4 (4)
C21—C22—H22	124.6	C16—C15—C14	114.9 (6)
C23—C22—H22	124.6	C16—C15—H15	122.5
C2—C3—C4	122.2 (5)	C14—C15—H15	122.5
C2—C3—H3	118.9	C15—C16—S2	113.6 (5)
C4—C3—H3	118.9	C15—C16—H16	123.2
C22—C21—C20	132.6 (6)	S2—C16—H16	123.2
C22—C21—S3	111.7 (4)		
			
O1—Al1—O3—C12	111.0 (4)	C16—S2—C13—C14	0.6 (4)
O4—Al1—O3—C12	15.6 (4)	C16—S2—C13—C12	−178.8 (4)
O5—Al1—O3—C12	−73.0 (4)	O1—C2—C1—F3	−85.8 (6)
O2—Al1—O3—C12	−158.2 (4)	C3—C2—C1—F3	96.5 (7)
O4—Al1—O5—C18	104.5 (4)	O1—C2—C1—F2	154.6 (5)
O3—Al1—O5—C18	−164.5 (4)	C3—C2—C1—F2	−23.0 (8)
O2—Al1—O5—C18	−76.5 (4)	O1—C2—C1—F1	35.0 (7)
O6—Al1—O5—C18	12.2 (4)	C3—C2—C1—F1	−142.7 (6)
O1—Al1—O2—C4	−8.8 (5)	C21—S3—C24—C23	0.5 (5)
O3—Al1—O2—C4	−99.2 (4)	Al1—O2—C4—C3	9.2 (8)
O5—Al1—O2—C4	168.3 (5)	Al1—O2—C4—C5	−170.9 (3)
O6—Al1—O2—C4	78.3 (4)	C2—C3—C4—O2	−4.9 (9)
O4—Al1—O1—C2	−174.4 (4)	C2—C3—C4—C5	175.2 (5)
O3—Al1—O1—C2	94.6 (4)	C5—C6—C7—C8	0.0 (7)
O2—Al1—O1—C2	6.4 (4)	O4—C10—C11—C12	−1.0 (8)
O6—Al1—O1—C2	−82.3 (4)	C9—C10—C11—C12	175.5 (5)
O1—Al1—O6—C20	161.0 (4)	O3—C12—C11—C10	−8.9 (8)
O4—Al1—O6—C20	−103.7 (4)	C13—C12—C11—C10	172.1 (5)
O5—Al1—O6—C20	−15.1 (4)	O5—C18—C17—F9	49.4 (7)
O2—Al1—O6—C20	70.1 (4)	C19—C18—C17—F9	−128.0 (6)
O1—Al1—O4—C10	−114.0 (4)	O5—C18—C17—F8	176.3 (5)
O3—Al1—O4—C10	−23.5 (4)	C19—C18—C17—F8	−1.2 (9)
O5—Al1—O4—C10	68.9 (4)	O5—C18—C17—F7	−65.0 (6)
O6—Al1—O4—C10	158.7 (4)	C19—C18—C17—F7	117.6 (6)
Al1—O3—C12—C11	−2.6 (7)	O5—C18—C19—C20	−2.8 (10)
Al1—O3—C12—C13	176.4 (3)	C17—C18—C19—C20	174.2 (5)
Al1—O5—C18—C19	−6.6 (8)	C6—C7—C8—S1	0.2 (7)
Al1—O5—C18—C17	176.3 (4)	C5—S1—C8—C7	−0.3 (5)
Al1—O4—C10—C11	21.0 (7)	S3—C24—C23—C22	−1.0 (7)
Al1—O4—C10—C9	−155.7 (3)	C21—C22—C23—C24	1.0 (8)
Al1—O1—C2—C3	−5.4 (8)	Al1—O6—C20—C19	11.1 (7)
Al1—O1—C2—C1	177.2 (4)	Al1—O6—C20—C21	−168.3 (3)
O4—C10—C9—F6	76.7 (5)	C18—C19—C20—O6	0.6 (8)
C11—C10—C9—F6	−100.4 (6)	C18—C19—C20—C21	−179.9 (5)
O4—C10—C9—F5	−42.8 (6)	C22—C21—C20—O6	−173.5 (6)
C11—C10—C9—F5	140.1 (5)	S3—C21—C20—O6	3.6 (6)
O4—C10—C9—F4	−162.2 (4)	C22—C21—C20—C19	7.0 (9)
C11—C10—C9—F4	20.8 (7)	S3—C21—C20—C19	−175.9 (4)
O1—C2—C3—C4	3.2 (9)	C7—C6—C5—C4	−178.5 (5)
C1—C2—C3—C4	−179.5 (5)	C7—C6—C5—S1	−0.2 (5)
C23—C22—C21—C20	176.7 (5)	O2—C4—C5—C6	−178.7 (5)
C23—C22—C21—S3	−0.5 (6)	C3—C4—C5—C6	1.2 (9)
C24—S3—C21—C22	0.0 (5)	O2—C4—C5—S1	3.0 (6)
C24—S3—C21—C20	−177.7 (4)	C3—C4—C5—S1	−177.1 (4)
C15—C14—C13—C12	177.7 (5)	C8—S1—C5—C6	0.3 (4)
C15—C14—C13—S2	−1.8 (6)	C8—S1—C5—C4	178.9 (4)
O3—C12—C13—C14	−179.1 (5)	C13—C14—C15—C16	2.4 (7)
C11—C12—C13—C14	−0.1 (8)	C14—C15—C16—S2	−2.0 (7)
O3—C12—C13—S2	0.3 (6)	C13—S2—C16—C15	0.8 (5)
C11—C12—C13—S2	179.3 (4)		
